# Ultra-low Thermal Conductivity in Si/Ge Hierarchical Superlattice Nanowire

**DOI:** 10.1038/srep16697

**Published:** 2015-11-16

**Authors:** Xin Mu, Lili Wang, Xueming Yang, Pu Zhang, Albert C. To, Tengfei Luo

**Affiliations:** 1 Department of Aerospace and Mechanical Engineering, University of Notre Dame, Notre Dame, IN 46556, USA; 2 Center for Sustainable Energy at Notre Dame, University of Notre Dame, Notre Dame, IN 46556, USA; 3 Department of Mechanical Engineering and Materials Science, University of Pittsburgh, Pittsburgh, PA 15261, USA; 4 School of Fundamental Studies, Shanghai University of Engineering Science, Shanghai, 201620, China; 5 Department of Power Engineering, North China Electric Power University, Baoding, 071003, China

## Abstract

Due to interfacial phonon scattering and nanoscale size effect, silicon/germanium (Si/Ge) superlattice nanowire (SNW) can have very low thermal conductivity, which is very attractive for thermoelectrics. In this paper, we demonstrate using molecular dynamics simulations that the already low thermal conductivity of Si/Ge SNW can be further reduced by introducing hierarchical structure to form Si/Ge hierarchical superlattice nanowire (H-SNW). The structural hierarchy introduces defects to disrupt the periodicity of regular SNW and scatters coherent phonons, which are the key contributors to thermal transport in regular SNW. Our simulation results show that periodically arranged defects in Si/Ge H-SNW lead to a ~38% reduction of the already low thermal conductivity of regular Si/Ge SNW. By randomizing the arrangement of defects and imposing additional surface complexities to enhance phonon scattering, further reduction in thermal conductivity can be achieved. Compared to pure Si nanowire, the thermal conductivity reduction of Si/Ge H-SNW can be as large as ~95%. It is concluded that the hierarchical structuring is an effective way of reducing thermal conductivity significantly in SNW, which can be a promising path for improving the efficiency of Si/Ge-based SNW thermoelectrics.

Thermoelectric materials have important applications in solid-state cooling, and direct conversion between thermal and electric energy^1^,^2^. In spite of the thermoelectric devices possess many unique advantages, the relatively low energy conversion efficiency remains a limiting factor to their competitiveness and the range of deployment^3^,^4^,^5^. The thermoelectric energy conversion efficiency is dictated by the dimensionless figure-of-merit, *ZT* = *S*^2^*σT*/*κ*, where *S*, *σ*, *T* and *κ* are Seebeck coefficient, electrical conductivity, absolute temperature and thermal conductivity of the material, respectively. Because these parameters are unavoidably inter-correlated to each other, increasing *ZT* is very challenging^6^,^7^,^8^,^9^,^10^,^11^. Significant scientific efforts have been dedicated to enhancing the *ZT* in the past decade by enhancing the Seebeck coefficients^12^,^13^,^14^,^15^,^16^, and reducing the lattice thermal conductivity^10^,^17^,^18^,^19^,^20^,^21^ while maintaining the carrier mobility^22^,^23^,^24^.

The thermal transport properties of silicon/germanium (Si/Ge)-based nanomaterials have been under intense investigations due to their potential applications in thermoelectric energy conversion^25^,^26^,^27^,^28^,^29^,^30^,^31^. It has been predicted by recent *ab-initio* calculations that the thermoelectric performance of Si/Ge-based nanomaterials can be improved by reducing the thermal conductivity with limited impact on their thermoelectric power factor (*S*^*2*^*σ*)^32^,^33^. Among the various Si/Ge-based nanomaterials, Si/Ge-based nanowires (NWs) are especially promising due to their inherent one-dimensionality, resulting in low thermal conductivity and simultaneous high carrier mobility^25^,^26^,^27^,^28^,^29^,^30^. The thermal transport properties of different Si/Ge-based NWs, such as Si/Ge alloy NWs^25^, Si/Si_1−x_Ge_x_ superlattice NWs^26^, Si_1−x_Ge_x_ NWs^27^, Si/Ge core-shell NWs^29^,^30^ have been investigated.

Due to the emergence of effective fabrication techniques, Si/Ge-based superlattice nanowires (SNWs) have now become very important candidates for thermoelectric applications^34^. It is well-known that the thermal conductivity of superlattice can be lower than its alloy counterparts due to the phonon scattering at superlattice interfaces and the reduction of phonon group velocity due to the formation of mini-bands in the phonon dispersion relation^28^,^35^,^36^,^37^,^38^,^39^. The Si/Ge-based SNWs take advantage of the size effect of NWs and characteristics of superlattice to achieve very low thermal conductivity. Using molecular dynamics (MD) simulation, Hu *et al.* found that the thermal conductivity of a Si/Ge SNW with perfect periodic structures can be one order of magnitude (92%) lower than that of a pure Si NW at room temperature^28^. Considering that the electrical transport properties of very thin superlattice nanowire do not change significantly compared to perfectly smooth NW, which is evidenced by recent *ab-initio* calculations^32^,^33^, this remarkable thermal conductivity reduction can be translated into almost an order of magnitude enhancement in the *ZT* coefficient.

The periodicity in superlattice structures can lead to the formation of coherent phonons, which is due to the constructive interference of incident and reflected phonon waves at the interfaces. Recent experiments on GaAs/AlAs superlattice^40^ and epitaxial perovskite oxide superlattice^41^ have shown that the coherent phonons can contribute significantly to the thermal transport in superlattice. A recent MD simulation study on isotopically modified graphene superlattice also confirmed the existence of coherent phonons in superlattice^36^. Using atomistic Green’s function method, Tian *et al.* studied the phonon transport in Si/Ge superlattice and demonstrated the existence of coherent phonons in this superlattice structure^42^. Using MD simulation, Hu *et al.* have also demonstrated the existence of coherent phonons in Si/Ge SNW when the periodic length is very short^28^. Moreover, some theoretical works have been performed to quantitatively study the coherent and incoherent phonon transport characteristics in superlattice^43^,^44^. Wang *et al.* built a two-phonon model that divides the overall heat conduction in superlattice into coherent and incoherent phonon contributions, and predicted the intrinsic mean free paths (MFPs) of both coherent and incoherent phonons^43^. Latour *et al.* introduced a microscopic definition of the phonon coherence length (CL), and a criterion was provided to distinguish the coherent transport regime from diffuse interface scattering^44^.

Now that it is understood that coherent phonons can contribute to the thermal transport in superlattice, strategies to scatter these coherent phonons or even prevent their formation will be valuable to further lowering thermal conductivity beyond those achieved using regular SNWs. Hierarchical structuring, which has been applied in thermoelectrics and photonics^20^,^45^, can be an effective way to further lower the thermal conductivity of Si/Ge SNW. Hierarchical structuring can significantly reduce thermal conductivity with little or no change to the thermoelectric power factor^20^. An experimental study of a hierarchical structured PbTe system has recently reported an unprecedented *ZT* value of 2.2^20^. The mechanism in their results is deduced to be that phonons of different wavelengths can be scattered by structures with different length scales. Hierarchical structuring can be a potentially effective way to reduce the thermal conductivity of superlattice if the imposed hierarchical length scales can scatter the coherent phonons in superlattice.

In this work, we use large scale MD simulations to systematically investigate the thermal transport in regular Si/Ge SNWs and Si/Ge hierarchical superlattice nanowires (H-SNWs). The thermal conductivities of Si/Ge H-SNWs are found to be much lower than those of the regular Si/Ge SNWs. The frequency dependent coherence lengths of regular Si/Ge SNWs and Si/Ge H-SNWs are calculated to quantitatively explain the coherent and incoherent phonon transport in superlattice. It is concluded that the hierarchical structures can not only scatter phonons by introducing structural defects but also impair the formation of coherent phonons due to the disruption of the periodicity of the SNWs. Additional structural complexities, such as amorphous surface and rough surface, are also found to further decrease thermal conductivity. The findings in this work can be very instrumental to improving the thermoelectric properties of Si/Ge-based SNW.

## Results and Discussion

### Thermal Transport in Pure Si NW, Pure Ge NW and Regular Si/Ge SNW

**Figure 1:**
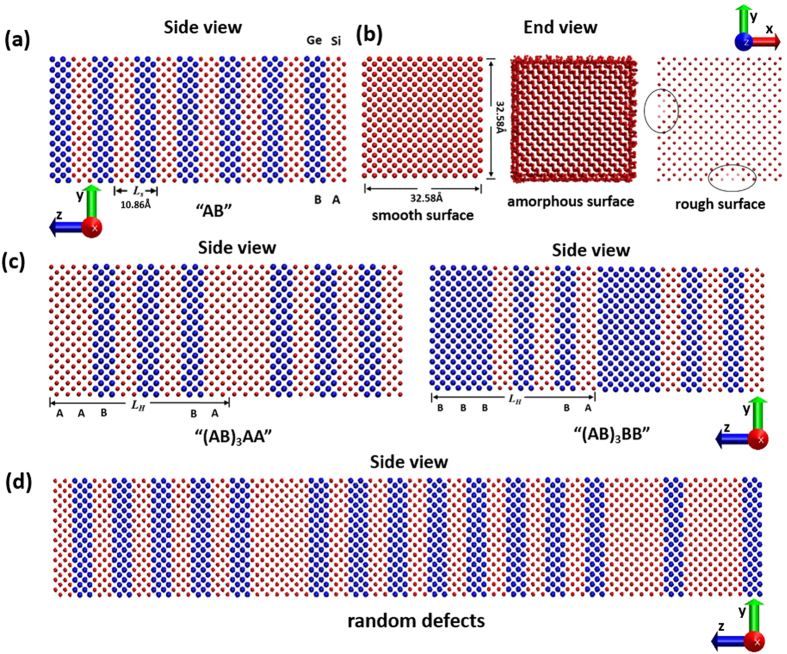
(**a**) The side view of sturcture of Si/Ge SNW with period of “AB”. (**b**) The end view of structures with different surface structures. Black circles in the rightmost panel highlight the regions with atoms removed to create surface roughness. (**c**) The side views of example structures of periodically defected Si/Ge H-SNWs with periods of “(AB)_3_AA” and “(AB)_3_BB”. (**d**) The side view of a Si/Ge H-SNW structure with randomly placed Si defects.

The regular Si/Ge SNW structures in our simulations are constructed by periodically stacking diamond-structured Si and Ge layers. The Si and Ge layers have the same size, and each of them contains 6 × 6 × *n* (32.58 × 32.58 × *n* • 5.43 Å^3^) unit cells in *x*, *y* and *z* directions. The lattice constant of the Ge layer is assumed to be the same as that of the Si layer in our simulation since epitaxial Si/Ge interface has shown to have small strain at the interface due to their relatively small lattice mismatch (~4%)^46^. One period of the Si/Ge SNW structure consists of one Si layer and one Ge layer. The period length is denoted as *L*_*s*_. We can change *n* and the number of total periods (*N*) to simulate Si/Ge SNWs with different period lengths and total lengths. Normal to the Si/Ge interface is the [011] crystal direction of the diamond-structured lattice. NW with this kind of surface orientation was experimentally found to be more stable^47^. To make discussion convenient, we use “A” to denote the Si layer (with thickness (in *z* direction) of 5.43 Å) and “B” to denote the Ge layer (with thickness (in *z* direction) of 5.43 Å) in the following text. Figure 1(a) shows the structure of regular Si/Ge SNW with period of “AB”. We study two kinds of Si/Ge H-SNWs: periodically Si or Ge defected SNW (denoted as H-SNW-PSi or H-SNW-PGe), and randomly Si or Ge defected SNW (denoted as H-SNW-RSi or H-SNW-RGe). Starting with regular SNW, H-SNW-PSi (or -PGe) is constructed by substituting one Si (or Ge) layer out of every few layers with a Ge (or Si) layer. The distance between these periodically placed structural defects is denoted as *L*_*H*_, and this distance is the period length of H-SNW-PSi (-PGe). The single period of the Si/Ge H-SNW-PSi and Si/Ge H-SNW-PGe are denoted as “(AB)_m_AA” and “(AB)_m_BB”, respectively. Subscript *m* means that there is one defect every *m* “AB” periods and we can change *m* to tune *L*_*H*_. Figure 1(c) shows the single period of two representative periodically defected Si/Ge H-SNWs with the periods of “(AB)_3_AA” and “(AB)_3_BB”. The structures of randomly defected SNW are achieved by randomizing the placements of Si or Ge defects in each single period of periodically defected Si/Ge H-SNW (see Fig. 1(d) for the structure). We also study the effects of amorphous surface and rough surface on thermal conductivity (see Fig. 1(b) for their structures). To achieve the amorphous surface, we fix the central portion of H-SNW and heat up the surface atoms to ~3000 K gradually, and then quench them down to 300 K. To roughen the H-SNW surface, we randomly remove some surface atoms from the H-SNW. In this study, we only investigate the thermal transport in the direction normal to the Si/Ge interface (*z* direction). Details of the simulation method are described in the Method section.

**Figure 2:**
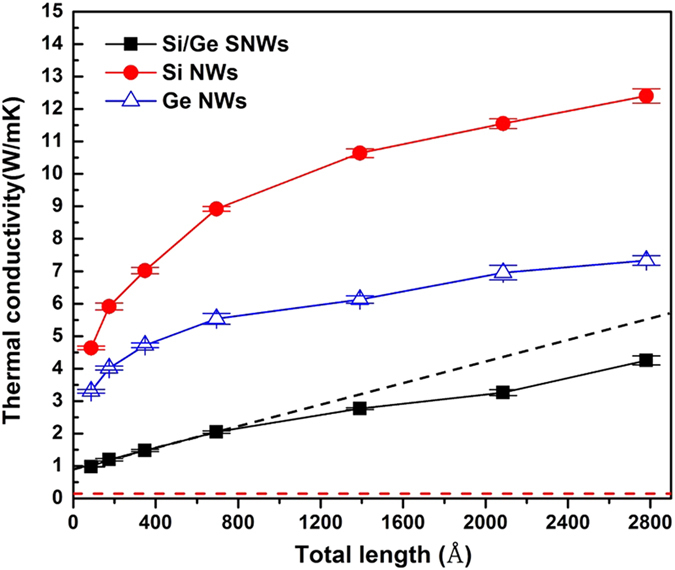
Thermal conductivity of pure Si NW, pure Ge NW and regular Si/Ge SNW with period of “AB” as a function of their total lengths. The red dashed line indicates the thermal conductivity of this regular Si/Ge SNW in the incoherent phonon transport limit. The black dash line shows the linear increase in thermal conductivity of regular Si/Ge SNW, which corresponds to the coherent phonon transport limit.

We first study the thermal transport in pure Si NW, pure Ge NW and regular Si/Ge SNW with period of “AB” to serve as references for Si/Ge H-SNW. Pure Si NW and Ge NW have the same struture as the Si and Ge layers in Si/Ge SNW. The thermal conductivities of pure Si NWs (red solid circles) and pure Ge NWs (blue hollow triangles) as a function of their total lengths are shown in Fig. 2. We can see that the thermal conductivities of pure Si NWs and Ge NWs both increase with their total lengths in the length range from 0 Å to 2780.2 Å, consistent with the study by Yang *et al.*^48^. This means that there are phonons transporting ballistically in these NWs with the length of sample being the limiting length for their MFPs. The trend of increasing thermal conductivity with total sample length is not unusual for common crystals when the boundary scattering dominates the phonon scattering processes – known as the classical size effect^49^. It is also seen that the thermal conductivities of pure Si and Ge NWs increase with their total lengths much slower than linear increasing, which means that not all phonons are ballistic but some of them are diffusive due to either phonon-surface scattering or the intrinsic phonon Umklapp scattering^50^,^51^,^52^,^53^. It has been reported that the thermal conductivities of pure Si and Ge NWs are diameter-dependent due to phonon-surface scattering even for the diameters as long as ~1000 Å^51^,^52^,^53^,^54^. The cross-sectional areas of the pure Si and Ge NWs in this work are both 32.58 × 32.58 Å^2^, thus there should be strong phonon-surface scatterings in the NWs that limit phonon’s MFPs. We extract the effective phonon MFP of pure Si NW and Ge NW based on the gray phonon model and find the values to be ~388.7 Å and ~313.6 Å, respectively (the extraction method is the same as the one used in Mu *et al.*’s paper^55^, and the extraction details are described in the Supporting Information (SI)). These values are much larger than their cross-sectional sizes, meaning that the NW surface scatterings are not completely diffusive, which would have decreased the MFP to the same order as the NW cross-sectional size. The thermal conductivities of pure Ge NWs are always smaller than those of pure Si NWs as long as they have the same total length, and both are much lower than their bulk counterparts.

In general, thermal transport in superlattice can be attributed to coherent and incoherent phonon transport. Coherent phonon modes preserve their phases as they propagate through multiple interfaces in the superlattice. These phonons, which are formed due to the constructive interference of incident and reflected phonon waves at the superlattice interfaces, can extend over the whole structure. In this picture, the superlattice can be regarded as a homogeneous material with its own unit cell and phonon dispersion. If only coherent phonon transport exists and the transport is ballistic, thermal resistance of the superlattice will remain constant regardless of the number of periods, and the thermal conductivity should increase linearly with the sample length. However, if phonons are scattered before being reflected at the interfaces or get diffuse scattering at the interfaces, these phonon modes will lose their phase coherence and the constructive interference at superlattice interfaces will not occur. Phonon transport becomes incoherent in this case. For those phonon modes, a superlattice acts as a composite made of stacks of alternating materials and interfaces, and the repeating units act as serial thermal resistors. In this extreme, the effective thermal conductivity of the superlattice is independent of the sample length. The effective thermal conductivity in the incoherent phonon transport extreme can be expressed as:


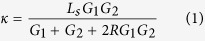


where *G*_*1*_ and *G*_*2*_ are the thermal conductance of each portion in a period of superlattice, *R* is the interfacial thermal resistance and *L*_*s*_ is the period length.

The thermal conductivity of regular Si/Ge SNW with *L*_*s*_ of ~10.86 Å as a function of their total lengths are shown in Fig. 2 (black solid squares). We can see that the thermal conductivities of regular Si/Ge SNWs increase almost linearly with their total length from 0 Å to ~695 Å. This indicates the existence of coherent phonon modes in this regular Si/Ge SNW, and these coherent phonons transport ballistically in this length range. In this case, the effective MFP of the coherent phonons is limited by the total length of the sample due to phonon scattering at the end boundaries. The linear increasing of thermal conductivity with total length has been observed experimentally and theoretically before, and was attributed to the coherent phonon transport^36^,^40^. For total length larger than ~695 Å, the increasing trend deviates from the linear relation. For these cases, the sample length becomes larger than the MFPs of some coherent phonons and thus their MFPs are determined by internal scattering events, including surface scattering and Umklapp scattering. However, the fact that the thermal conductivity continues to increase, although slower than the linear trend, indicates that there are still some coherent phonons transporting ballistically. We extract the effective MFP of this regular Si/Ge SNW based on the gray phonon model and obtain a value of ~1169.2 Å (see calculation details in SI). The effective MFP is larger than the upper limit of the coherent phonons ballistic transport length range (~695 Å), which confirms the conclusion we have drawn above. It is also noted that the effective MFP of this regular Si/Ge SNW is even larger than the effective MFPs of pure Si and Ge NWs (~388.7 Å and ~313.6 Å respectively). This means the coherent phonons have weaker phonon Umklapp scattering than phonons in Si and Ge NWs. This also demonstrates that the Si/Ge SNW has smaller phonon group velocity than Si and Ge NWs, as it has smaller thermal conductivity than pure Si and Ge NWs regardless of the total length, which is shown in Fig. 2. The effective phonon group velocities can also be fitted from the data in Fig. 2 (see SI for details), and the values we obtain are ~1031.8 ms^−1^, ~731.6 ms^−1^ and ~133.6 ms^−1^ for pure Si NW, pure Ge NW and regular Si/Ge SNW, respectively. The smaller phonon group velocity of Si/Ge SNW is due to the formation of mini-bands in its phonon dispersion relation.^28^,^35^,^36^,^37^,^38^,^39^

The basis of the above observations in superlattice is the existence of coherent phonons. Otherwise, the thermal conductivity should be a constant as expressed by Equation (1). We calculate the thermal conductivity of the regular Si/Ge SNW discussed above in its incoherent phonon transport extreme according to Equation (1), and obtain a value of ~0.14 Wm^−1^ K^−1^, which is shown by the red dashed line in Fig. 2 (see calculation details in SI). We can see that the thermal conductivity of the regular Si/Ge SNW is always larger than that in its incoherent phonon transport extreme, which demonstrates the coherent phonon transport exists in the Si/Ge SNW. The existence of coherent phonons can also be indicated by a minimum in thermal conductivity when *L*_*s*_ is changed^38^,^44^,^56^. The minimum thermal conductivity can also indicate the crossover from coherent to incoherent phonon transport. We studied the thermal conductivity of regular Si/Ge SNW as a function of its *L*_*s*_, and the results are discussed in the SI, Fig. S2.

The formation of coherent phonons in this Si/Ge SNW can be attributed to two reasons. The first one is that the Si/Ge SNW has very small period length (~10.86 Å), which is much smaller than the effective MFPs of pure Si NW (~388.7 Å) and Ge NW (~313.6 Å). Within such small period, most phonons will not be scattered by internal phonon-phonon Umklapp scattering or phonon-surface scattering, and their phases can be preserved when they propagate to the Si/Ge interfaces. The second reason is that the Si/Ge SNW structures have perfect epitaxial Si/Ge interfaces, and the phonons can have specular interface scattering, which allows phonons to maintain their phases. These two reasons enable the incident and reflected phonon waves to have phase coherence and interfere constructively at the interfaces to form coherent phonons.

**Figure 3:**
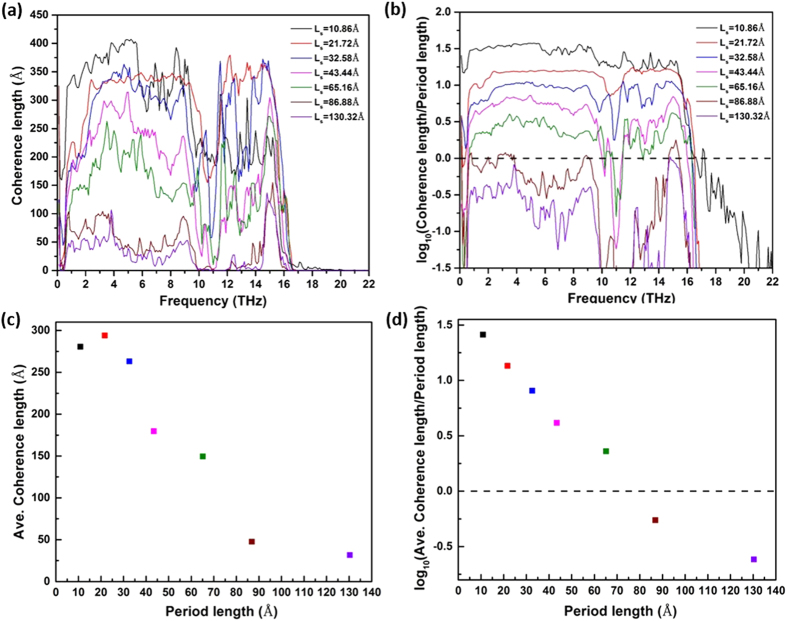
(**a**) Phonon CL of regular Si/Ge SNWs with different period lengths, *L*_*s*_, as a function of the phonon frequency. (**b**) log_10_(CL/*L*_*s*_) as a function of the phonon frequency. The black horizontal dash line indicates the threshold of phonons with CLs larger or smaller than the *L*_*s*_. (**c**) DOS weighted average of CL, CL_ave_, as a function of *L*_*s*_. (**d**) log_10_(CL_ave_/*L*_*s*_) as a function of *L*_*s*_. The black horizontal dash line also indicates the threshold of phonons with CLs larger or smaller than the *L*_*s*_.

Phonon CL is an important quantity to characterize the coherent and incoherent phonon transport in superlattice. If CL is larger than the superlattice *L*_*s*_, which means that the spatial extension of phonon wave-packet is larger than *L*_*s*_, the phonon transport is coherent; while on the opposite, the phonon transport is incoherent^44^. According to Latour *et al.*, phonon CL can be extracted from atomic trajectories^44^. Using this method, we calculate the frequency- dependent phonon CL of regular Si/Ge SNW with different *L*_*s*_, as shown in Fig. 3(a) (calculation details in SI). Overall, the CL decreases with *L*_*s*_. As *L*_*s*_ increases, the larger period lengths provide longer distance for intrinsic phonon-phonon and phonon-surface scatterings to happen, which largely impair the possibility of forming coherent phonons at the interfaces, and thus the phonon CL becomes smaller. Figure 3(b) shows log_10_(CL/*L*_*s*_) as a function of phonon frequency. We can see that for the cases with *L*_*s*_in the range of 10.86 Å to 32.58 Å, the log_10_ (CL/*L*_*s*_) values are almost completely positive, which means the CLs for the whole phonon spectrum are larger than the period length. Thus, thermal transport in Si/Ge SNWs with these period lengths is coherent phonon dominant. Such an observation generally agrees with the thermal conductivity trend shown in Figure S2 in SI. For the cases with *L*_*s*_ ranging from 32.58 Å to 65.16 Å, the log_10_(CL/*L*_*s*_) values are partially negative, which means the CLs can be smaller than *L*_*s*_ depending on the phonon frequencies. In this range, the transition from coherent to incoherent phonon transport is believed to be happening. When *L*_*s*_ is larger than 65.16 Å, all phonons have CLs smaller than the *L*_*s*_ and the thermal transport is dominated by the incoherent phonons. For a clearer comparison, we convolute the CLs with the phonon density of states (DOS) and obtain a single value for each *L*_*s*_ (Fig. 3(c,d)) (calculation details in SI). It can be seen that the average CLs are larger than *L*_*s*_ until the *L*_*s*_ is larger than 65.16 Å. Such information can guide us to place the defects in Si/Ge SNW to impair the formation of coherent phonons or scatter the coherent phonons, and thus reducing the thermal conductivity.

### Thermal Transport in Si/Ge H-SNW

**Figure 4:**
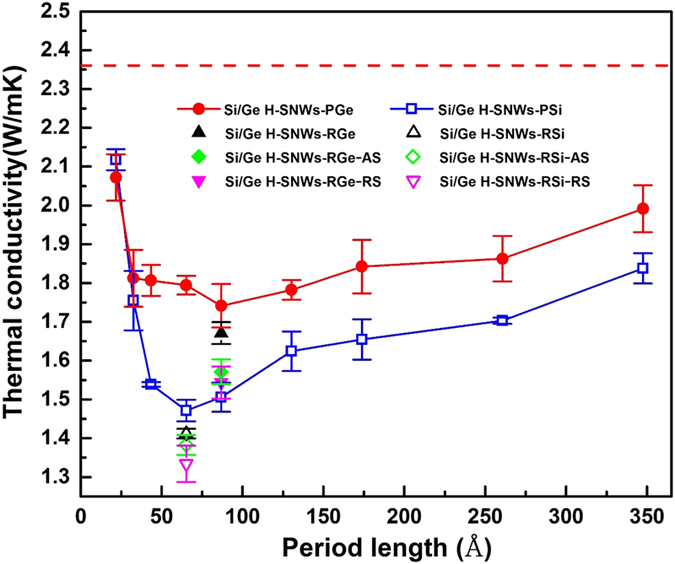
Thermal conductivities of Si/Ge H-SNW-PSi and Si/Ge H-SNW-PGe as a function of their period lengths, *L*_*H*_. The thermal conductivities of Si/Ge H-SNW-RSi, Si/Ge H-SNW-RGe, Si/Ge H-SNW-RSi with amorphous and rough surfaces (Si/Ge H-SNW-RSi-AS and Si/Ge H-SNW-RSi-RS), and Si/Ge H-SNW-RGe with amorphous and rough surfaces (Si/Ge H-SNW-RGe-AS and Si/Ge H-SNW-RGe-RS) are also shown here for comparison. The red dash line represents the thermal conductivity of regular Si/Ge SNW with period of “AB”. All the cases have the same total length: ~1042.6 Å.

From the discussion so far, we understand that a large portion of the thermal conductivity in regular SNW, especially for the cases with small period lengths, is contributed by coherent phonons. It is thus possible to reduce the thermal conductivity by either hampering the formation of coherent phonons or scattering the coherent phonons. This can be achieved by using hierarchical structuring, which introduces structural defects into the superlattice. Figure 4 shows the thermal conductivity of periodically defected Si/Ge H-SNW as a function of their period length, *L*_*H*_. For periodically defected H-SNWs, they have periodicity with repeating units of “(AB)_m_AA” or “(AB)_m_BB” for Si or Ge defected cases. The blue hollow squares in Fig. 4 represent the thermal conductivities of Si/Ge H-SNW-PSi and the red solid circles represent those of Si/Ge H-SNW-PGe. The total lengths of all the H-SNWs are ~1042.6 Å. The red horizontal dash line indicates the thermal conductivity of regular Si/Ge SNW with period of “AB” and the same total length as the Si/Ge H-SNWs.

From Fig. 4, we can see that the thermal conductivities of periodically defected Si/Ge H-SNWs are always smaller than that of regular Si/Ge SNW (shown by red dash line). The reasons can be two folds. On one hand, when *L*_*H*_ is smaller than the coherent length of the regular Si/Ge SNW, the defects can disrupt the periodicity of the regular Si/Ge SNW, and thus impair the formation of the coherent phonons. For the regular Si/Ge SNW with the period of “AB”, the DOS weighted average of CL (shown in Fig. 3(c)) is ~280 Å, and thus most of the H-SNWs studied in Fig. 4 benefit from this factor. On the other hand, the CL of coherent phonons with different frequencies spans a large range (see Fig. 3). For coherent phonons with CLs much smaller than *L*_*H*_, the defects can work as scattering centers for the coherent phonons to reduce their MFPs. We calculate the effective phonon MFPs based on the gray phonon model for periodically defected H-SNWs with two different *L*_*H*_: *L*_*H*_ = 21.72 Å (the corresponding periods are “ABAA” and “ABBB”) and *L*_*H*_ = 347.52 Å (the corresponding periods are “AB31AA” and “AB31BB”). For *L*_*H*_ = 21.72 Å, the effective phonon MFPs of “ABAA” and “ABBB” cases are ~688.5 Å and ~403.5 Å, respectively. We can see that the effective phonon MFPs of these two H-SNWs are smaller than that of the regular Si/Ge SNW (~1169.2 Å). For *L*_*H*_ = 347.52 Å, the effective phonon MFPs of “AB31AA” and “AB31BB” cases are ~496.9 Å and ~535.6 Å, which are also smaller than that of regular Si/Ge SNW (~1169.2 Å). The calculation details of MFPs can be found in the SI.

For both Si/Ge H-SNW-PSi and Si/Ge H-SNW-PGe, there are also minimum thermal conductivities as *L*_*H*_ changes. This means that the periodically defected H-SNWs can form their own coherent phonons with “(AB)_m_AA” or “(AB)_m_BB” being the period. Considering the minimum thermal conductivities, the Si/Ge H-SNW-PSi leads to a ~38% reduction in thermal conductivity compared to the regular Si/Ge SNW with the same total length (shown by the red dash line in Fig. 4), and the H-SNW-PGe shows a ~26% reduction. It is interesting to see that the H-SNW-PGe shows lower thermal conductivity than H-SNW-PSi in almost all *L*_*H*_ studied here. This is counter-intuitive since Ge is a heavier atom than Si, which should lead to a smaller phonon group velocity and thus lower thermal conductivity. One possibility is that their coherent phonons may be different. We calculate the DOS weighted average of the CLs of H-SNW-PSi and H-SNW-PGe with *L*_*H*_ = 65.16 Å (calculation details in SI), and obtain values of ~267.5 Å and ~284.8 Å, respectively. The fact that H-SNW-PGe has longer phonon CL than H-SNW-PSi could be one reason for the higher thermal conductivity of H-SNW-PGe.

It is worth noting that for the Si/Ge H-SNWs discussed above, periodicity still exists in the structure but with different period lengths compared to regular Si/Ge SNW. We add another kind of hierarchy by randomizing the placement of defects in the Si/Ge H-SNW and thus completely eliminate the long-range periodicity. To achieve this, we take the samples with periods of “(AB)_5_AA” and “(AB)_7_BB”, which correspond to the cases with the minimum thermal conductivities of Si/Ge H-SNW-PSi and H-SNW-PGe, respectively, and randomize the locations of defects in each period (see Fig. 1(d) for the structure). As mentioned above, we denote these two cases as Si/Ge H-SNW-RSi and H-SNW-RGe. The thermal conductivities of these two cases are shown by black hollow and solid up triangles respectively in Fig. 4. We can see that the thermal conductivities of these two cases are smaller than the respective minimum thermal conductivities of Si/Ge H-SNW-PSi and H-SNW-PGe.

To further decrease the thermal conductivity, we add two kinds of complexities to the Si/Ge H-SNW-RSi and H-SNW-RGe: amorphous surface and rough surface (see Fig. 1(b) for their structures). These two complexities are supposed to increase the surface-phonon scattering and thus decrease the thermal conductivity^50^,^57^,^58^. The thermal conductivities of the amorphous and rough surface cases are shown in Fig. 4, and further decreased values are observed (the reduction of thermal conductivity can be as large as ~11%).

It is also observed that the thermal conductivities of H-SNWs with rough surfaces are smaller than those with amorphous surfaces. When atoms are removed from the surface, the effective cross-sectional sizes of the H-SNWs are smaller than those of the rough surface cases. This further reduces the limiting length scale posed by the small cross-sectional size and thus leads to smaller phonon MFPs. To quantitatively explain this, we calculate the effective MFPs of Si/Ge H-SNW-RSi, Si/Ge H-SNW-RSi with amorphous surface and Si/Ge H-SNW-RSi with rough surface, and obtain the values as ~724.0 Å, ~329.1 Å and 216.5 ~ Å respectively. We can see that the amorphous and rough surfaces can effectively decrease the phonon MFP, and the rough surface case has smaller phonon MFP than the amorphous surface case. The same trend is also found for Ge-defected cases (~708.3 Å, ~652.3 Å and ~522.8 Å for respective cases). Considering that the thermal conductivity of regular Si/Ge SNW is only ~8% of that of pure Si NW as predicted by Hu *et al.*^28^, we calculate the overall thermal conductivity reduction of Si/Ge H-SNW compared to the pure Si NW, and find the reduction is as large as ~95%. This is very desirable for the thermoelectric application.

## Conclusions

In summary, through MD simulations we find that a large portion of the thermal conductivity in regular Si/Ge SNW is contributed by coherent phonons. By perturbing the periodicity of the superlattice and thus impair the formation of coherent phonons, the thermal conductivity of Si/Ge SNW can be decreased. The Si/Ge H-SNW with periodic Si defects can achieve a ~38% reduction in thermal conductivity compared to regular Si/Ge SNW, and that with periodic Ge defects can enable a ~26% reduction. By randomizing the placement of defects, further reduction in thermal conductivity can be achieved. By amorphizing and roughening the surface of Si/Ge H-SNW, further thermal conductivity reductions (by as large as ~11%) are also observed. Overall, compared to pure Si NW, the thermal conductivity reduction of Si/Ge H-SNW can be as large as ~95%. It is concluded that the hierarchical structuring is an effective way to reduce the thermal conductivity of Si/Ge SNW, which can serve as a guidance to improve the energy conversion efficiency of Si/Ge-based thermoelectrics.

## Method

All the MD simulations in this work are performed using the large-scale atomic/molecular massively parallel simulator (LAMMPS)^59^. Large vacuum spaces are maintained in the *x* and *y* directions to eliminate the interaction between the SNW with its images to simulate free surfaces. Periodic or isolated boundary conditions are used in the *z* direction depending on different steps in the simulation procedure. The Tersoff potential with the original parameters optimized for Si-Ge systems^60^ is used. This potential has been used to predict the thermal transport properties of Si NWs with amorphous layer^57^, nanoporous SiGe^61^, Si-Ge quantum dot superlattices^62^, crystalline Si/Ge nano-composites^63^ and Si/Ge SNWs^28^ very well. The total energies of all simulation systems are minimized using the steepest descent algorithm^64^ before the systems are simulated at 300 K in a constant number of atoms, pressure and constant temperature ensemble (NPT). For the NPT ensemble, the Nose-Hoover method^65^,^66^ is used for the thermostating/barostatting and the pressure in *z* direction is set to be 1 atm. During this optimization process, periodic boundary conditions are used in *z* direction. After reaching the equilibrium state, the equilibrium trajectories (position and velocity of each atom) of 20 ps are stored for calculating the phonon CL of Si/Ge SNWs and H-SNWs. The details of calculating the phonon CL can be found in SI.

Based on the optimized structure obtained, we use the non-equilibrium MD (NEMD) method to calculate the thermal conductivity. The method is similar to those used in ref. [28,^36^]. We fix the atoms at the ends of the structure in the *z* direction and run the simulations in a constant number of atoms, volume and constant temperature ensemble (NVT) for 1 ns to equilibrate the system. We then impose a temperature gradient across the simulation domain by setting the temperatures of the two ends at different values using Langevin thermostats^67^. The temperature difference between the two ends are 30 K. Except for the fixed ends, the rest of the atoms are simulated in a constant number of atoms, volume and constant energy ensemble (NVE). Isolated boundary conditions are used in the *z* direction in these simulations. Due to the temperature difference, heat flows from the heat source to the heat sink across the sample. Energies added into the heat source and subtracted from the heat sink are recorded. When steady state is reached, the temperature gradient (*dT/dx*) is obtained by fitting the linear portion of temperature profile, and the heat flux (*J*) is calculated using:


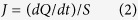


where *dQ/dt* is the average of the energy input and output rates in the thermostated regions, and *S* is the cross-sectional area. In this work, all the samples have the same cross-sectional area: 32.58 × 32.58 Å^2^. To reach steady state, an NVE run for 5 ns is usually needed. The thermal conductivity (*κ*) is then calculated using Fourier’s law of heat conduction:


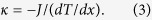


For each simulation, four thermal conductivity values are obtained from four different time blocks in the steady state, and the final value is the average of these four values with the error bar being the standard deviation. A time step of 1 fs is used for all the simulations.

It is noted that all the simulations in this work are classic MD simulations without any quantum corrections implemented. For Si-Ge nanocomposites, it was found the quantum correction could influence the results^68^. However, the only thing quantum correction does is correcting the temperature according to Bose-Einstein distribution^69^. Since our simulations are all at the same temperature, the physics extracted should still be valid.

## Additional Information

**How to cite this article**: Mu, X. *et al.* Ultra-low Thermal Conductivity in Si/Ge Hierarchical Superlattice Nanowire. *Sci. Rep.* **5**, 16697; doi: 10.1038/srep16697 (2015).

## Supplementary Material

Supporting Information
