# Corrigendum: Ultra-low Thermal Conductivity in Si/Ge Hierarchical Superlattice Nanowire

**DOI:** 10.1038/srep32904

**Published:** 2016-09-14

**Authors:** Xin Mu, Lili Wang, Xueming Yang, Pu Zhang, Albert C. To, Tengfei Luo

Scientific Reports 5: Article number: 1669710.1038/srep16697; published online: 11 16 2015; updated: 09 14 2016

The original Supplementary Information file published with this Article was incorrect. This version included errors in the ‘Calculation of Phonon Coherent Length (CL) in Si/Ge SNW and H-SNW’ section and a number of typographical errors. The correct Supplementary Information file now accompanies the Article.

