# Supplementary material for: Ultra-low Thermal Conductivity in Si/Ge Hierarchical Superlattice Nanowire
Source: Sci Rep. 2015 Nov 16;5:16697. doi: 10.1038/srep16697 (PMC4644949; doi:10.1038/srep16697)

Supporting Information

Ultra-low Thermal Conductivity in Si/Ge Hierarchical Superlattice Nanowire

Xin Mu[1], Lili Wang[3,4], Xueming Yang[5], Pu Zhang[3], Albert C. To[3], Tengfei Luo[1,2]

[1] Department of Aerospace and Mechanical Engineering, University of Notre Dame, Notre Dame, IN 46556,USA

[2] Center for Sustainable Energy at Notre Dame, University of Notre Dame, Notre Dame, IN 46556, USA

[3] Department of Mechanical Engineering and Materials Science, University of Pittsburgh, Pittsburgh, PA 15261, USA

[4] School of Fundamental Studies, Shanghai University of Engineering Science, Shanghai, 201620, China

[5] Department of Power Engineering, North China Electric Power University, Baoding, 071003, China

1. Extracting Mean Free Path (MFP) Using the Gray Model

If we assume that all the phonons have the same group velocity and relaxation time (the gray phonon model approximation), it can be shown, through the Boltzmann transport equation and the Matthiessen's rule, that the inverse of thermal conductivity ($1/\kappa$) can be expressed as:[1]

$$\frac{1}{\kappa} = \frac{3}{cv}\left(\frac{1}{l_{ph}} + \frac{1}{l_b}\right) \tag{s1}$$

where c is the volumetric heat capacity, v is the effective phonon group velocity, l_{ph} is the effective intrinsic phonon MFP in the infinitely long limit, and l_b is the phonon free path due to boundary scattering, which is the sample length (L) along the heat transport direction. For the silicon/germanium (Si/Ge) superlattice nanowire (SNW) in our classic molecular dynamics (MD) simulation, c, v and l_{ph} are constant. Thus, $1/\kappa$ and $1/l_b$ have a linear relationship. When we plot $1/\kappa$ against $1/L$, we can obtain l_{ph} and v by linearly fitting the data. It has been found that such a fitting is only valid when the sample lengths are on the same order as the l_{ph}.[2] Figure S1 shows the data and linear fitting of $1/\kappa$ against $1/L$ for Si nanowire (NW). These four data points in Figure S1 correspond to the four longest cases of Si NW in Figure 2 in main text. The l_{ph} of Si NW we get from this linear fitting is ~388.7Å, and v we get is ~708.3 ms^{-1}. The l_{ph} and

v of other NWs discussed in the main text are obtained using the same method. It is noted that we use the classic definition of c (1.035×10^{6} $Jm^{-3}K^{-1}$) for the calculation of v.

2. Thermal Conductivity of Regular Si/Ge SNW at Incoherent Limit

To obtain the thermal conductivity in the incoherent limit of regular Si/Ge SNW with period length of 10.86 Å according to Equation (1) in the main text, we need to calculate the thermal conductance of Si and Ge portions (each has length of 5.43 Å) in one period, and the interfacial thermal resistance at the Si/Ge interface. As the Si and Ge NWs' thermal conductance are approximately constant as long as their total lengths are much less than their effective MFPs, we calculate the thermal conductance of Si and Ge NWs with total lengths of ~84 Å and use these values as the thermal conductance of Si and Ge portions in Si/Ge SNW with period length of 10.86 Å. It is noted that the effective MFPs of Si and Ge NWs are ~388.7 and ~313.6 Å respectively, which makes this calculation valid. The thermal conductance of Si and Ge NWs with total length of ~84 Å is calculated by dividing their respective thermal conductivities by their total lengths, and we obtain the values of ~564.1 and ~385.8 $MWm^{-2}K^{-1}$ respectively. We perform a separate NEMD calculation on a single Si/Ge interface and obtain a value of 1.6×10^{-9} $m^{2}KW^{-1}$ for the interfacial thermal resistance. In this calculation, the lengths of Si and Ge portions are both 172.5 Å. The calculation method for this single Si/Ge interface thermal resistance is very similar to the one used in Luo *et al.*'s paper.[3] We note that these calculations will likely contain some errors due to size effect on both thermal conductivity and interfacial thermal conductance. However, we do not expect the order of magnitude to differ.

3. Crossover of Coherent and Incoherent Phonon Transport in Si/Ge SNW

According to Simkin *et al.*, in a superlattice, there should be a crossover from coherent to incoherent phonon transport when its period length (L_S) increases, and such a transition can be indicated by the existence of a minimum thermal conductivity as a function of L_S.[4-6] Figure S2 shows the thermal conductivity of Si/Ge SNW as a function of L_S. All the samples in this figure have the same total length of ~1042.6 Å. We can see that the thermal conductivity decreases when L_S increases from 10.86 to 32.58 Å and increases afterwards, showing a minimum thermal conductivity when L_S is 32.58 Å. The value of this minimum thermal conductivity is 1.81 $Wm^{-1} K^{-1}$, which is only ~18.8% and ~29.8% of the thermal conductivities of pristine Si and Ge NWs with the same total length. This large thermal conductivity reduction in Si/Ge SNW is consistent with the one reported by Hu *et al.*.[7]

The minimum thermal conductivity in the superlattice indicates the situation where the phonon transport crosses over from coherent to incoherent phonon transport. To the left of the minimum, the small period length allow phonons to transport ballistically before getting reflected at the interface, thus have higher probability of forming coherent phonons. In this region, the coherent phonons are dominant. The thermal conductivity decreases with L_S is believed due to the decrease of phonon group velocity.[6] As L_S increases, more Brillouin zone folding is present. This leads to a larger number of mini-bands in the phonon dispersion relation, and thus reducing phonon group velocity.

To the right of the minimum, the large period length provides enough distance for intrinsic phonon-phonon and phonon-surface scatterings, which largely impairs the possibility of forming coherent phonon modes over multiple periods. In this region, incoherent phonons are dominant in the superlattice. As a result, when the period length increases, the interface density decreases and the smaller number of interfaces presents lower total thermal resistance, and thus the thermal conductivity of the superlattice increases.

4. Calculation of Phonon Coherent Length (CL) in Si/Ge SNW and H-SNW

Latour *et al.* introduced a microscopic definition of the phonon CL, and the technique to extract phonon CL from MD simulation was also provided.[5] Here, we reproduce some important steps of this technique. In general, the velocity field of crystal atoms can be defined as $\vec{v}(\vec{r}_i^{0\gamma},t)$, where $\vec{r}_i^{0\gamma}$ is the equilibrium position of atom γ in unit cell *i*. The atomic positions can be written as:

$$\vec{r}_i^{0\gamma} = \vec{r}_i^{0} + \vec{r}^{\gamma} \tag{s2}$$

where $\vec{r}_i^{0}$ is the position of cell *i* at equilibrium, and $\vec{r}^{\gamma}$ is the position of atom γ in this cell. The basis set used is orthogonal basis (see Figure S3 (a)). $\vec{e}_{||}$ is in the direction along which the correlation is done, $\vec{e}_{\perp 1}$ and $\vec{e}_{\perp 2}$ are in the directions orthogonal to $\vec{e}_{||}$. The mutual coherence function, $\Gamma^{\alpha\beta}(z_m, z_n, \tau)$, can be defined as:

$$\begin{aligned}\Gamma^{\alpha\beta}(z_m, z_n, \tau) = \frac{1}{k_B T N_\perp}\sum_{i=1}^{N_c}\sum_{j=1}^{N_c}\sum_{\gamma=1}^{N_b}\sqrt{m_i^\gamma m_j^\gamma}\left\langle v^\alpha(\vec{r}_i^{0\gamma},t)v^\beta(\vec{r}_j^{0\gamma},t+\tau)\right\rangle \delta[(\vec{r}_i^{0}-\vec{r}_j^{0})\cdot\vec{e}_{\perp 1}] \\ \delta[(\vec{r}_i^{0}-\vec{r}_j^{0})\cdot\vec{e}_{\perp 2}]\delta[\vec{r}_i^{0}\cdot\vec{e}_{||}-z_m]\delta[\vec{r}_j^{0}\cdot\vec{e}_{||}-z_n]\end{aligned} \tag{s3}$$

where k_B is the Boltzmann constant, *T* is the system temperature, $N_\perp$ is the number of cells in the orthogonal plane to $\vec{e}_{||}$, N_c is the total number of cells in the crystal, N_b is the number of atoms in the cell basis, m_i^γ is the mass of atom γ in the cell *i*, m_j^γ is the mass of atom γ in the cell *j*, $\delta[x]$ is the Dirac function, and the superscripts α and β are two components of the vector field. It is noted that $(m, n) \in \{1, N_{||}\}^2$, where $N_{||}$ is the number of the cells along $\vec{e}_{||}$. This mutual coherence function characterizes the mutual coherence between two transverse planes along $\vec{e}_{||}$ with coordinates z_m and z_n. The core part of the $\Gamma^{\alpha\beta}(z_m, z_n, \tau)$ is the sum of time correlations

of velocity fields between all pairs of atoms separated by the distance of $\left|z_m - z_n\right|$. In the triple sum, the atoms i and j belong to the atomic planes which locates at z_m and z_n respectively.

When the Fourier transform is taken on Equation (s3), the two-point cross-spectral density function $W(z_m, z_n, \omega)$ can be obtained. For each frequency ω, $W(z_m, z_n, \omega)$ characterizes the space-dependent correlation along $\vec{e}_{\|}$. The degree of coherence $\mu(z_m, z_n, \omega)$ then can be expressed as:

$$\mu(z_m, z_n, \omega) = \frac{W(z_m, z_n, \omega)}{[W(z_m, z_m, \omega)]^{1/2}[W(z_n, z_n, \omega)]^{1/2}} \quad \text{(s4)}$$

In this expression, the superscripts α and β are removed for simplicity. Also, $W(z_m, z_m, \omega)$ corresponds to the density of states (DOS) of all atoms at $z = z_m$, and thus we can obtain the DOS of every atomic plane.

In the following, spatial cross-correlation $C(k\Delta z, \omega)$ can be defined as:

$$C(k\Delta z, \omega) = \frac{1}{N_{\|} - k} \sum_{i=1}^{N_{\|}-k} \mu(z_i, z_{i+k-1}, \omega) \quad \text{(s5)}$$

Here $k \in \{0 ... N_{\|} - 1\}$, and Δz is the spatial resolution of the correlation. In my work, as $\vec{e}_{\|}$ is collinear to z direction (shown in Figure S3 (a)), Δz corresponds to the lattice parameter along this direction. At last, the spatial phonon CL ($l_c(\omega)$) can be extracted from the variance of the normalized cross-spectral density function as:

$$l_c^2(\omega) = \frac{\sum_{k=0}^{N_{\|}-1} \left|C(k\Delta z, \omega)\right|^2 (k\Delta z)^2}{\sum_{k=0}^{N_{\|}-1} \left|C(k\Delta z, \omega)\right|^2} - \left(\frac{\sum_{k=0}^{N_{\|}-1} \left|C(k\Delta z, \omega)\right|^2 (k\Delta z)}{\sum_{k=0}^{N_{\|}-1} \left|C(k\Delta z, \omega)\right|^2}\right)^2 \quad \text{(s6)}$$

For the MD simulation, we first run a constant pressure and constant temperature ensemble (NPT) for 2ns to relax the structure, and then run a constant volume and constant temperature ensemble (NVT) for 1ns and a constant volume and constant energy ensemble (NVE) for another 1ns to achieve the equilibrium state. We then continue to run the NVE ensemble, and

the equilibrium trajectories (position and velocity of each atom) of 20 ps are stored for calculating the CL. We then follow the equations given above calculating the frequency dependent CL. The phonon DOS can be calculated according to $W(z_m, z_m, \omega)$. Figure S3 (b) and (c) show the frequency-dependent CL and DOS of Si/Ge SNW with period of “AB”. The DOS of Si/Ge SNW is the mass weighted average of the DOSs of Si and Ge planes in Si/Ge SNW. We do the DOS weighted average on the frequency-dependent CL, and obtain a single value of ~280 Å for this case. The same method is used for obtaining the frequency-dependent CL, DOS and DOS weighted average of CL for all other Si/Ge SNWs and H-SNWs.

Reference

1. Schelling, P. K., Phillpot, S. R. & Keblinski, P. Comparison of atomic-level simulation methods for computing thermal conductivity. *Phys. Rev. B* **65**, 144306 (2002).

2. Sellan, D. P., Landry, E. S., Turney, J. E., McGaughey, A. J. H. & Amon, C. H. Size effects in molecular dynamics thermal conductivity predictions. *Phys. Rev. B* **81**, 214305 (2010).

3. Luo, T. & Lloyd, J. R. Molecular dynamics study of thermal transport in GaAs-self-assembly monolayer-GaAs junctions with ab initio characterization of thiol-GaAs bonds. *J. Appl. Phys.* **109**, 034301 (2011).

4. Chen, Y., Li, D., Lukes, J. R., Ni, Z. & Chen, M. Minimum superlattice thermal conductivity from molecular dynamics. *Phys. Rev. B* **72**, 174302 (2005).

5. Latour, B., Volz, S. & Chalopin, Y. Microscopic description of thermal-phonon coherence: From coherent transport to diffuse interface scattering in superlattices. *Phys. Rev. B* **90**, 014307 (2014).

6. Simkin, M. V. & Mahan, G. D. Minimum Thermal Conductivity of Superlattices. *Phys. Rev. Lett.* **84**, 927-930 (2000).

7. Hu, M. & Poulikakos, D. Si/Ge superlattice nanowires with ultralow thermal conductivity. *Nano Lett.* **12**, 5487-5494 (2012).

Figure S1. 1/κ as a function of 1/L for Si NW. The black dash line is the linear fitting of the data.

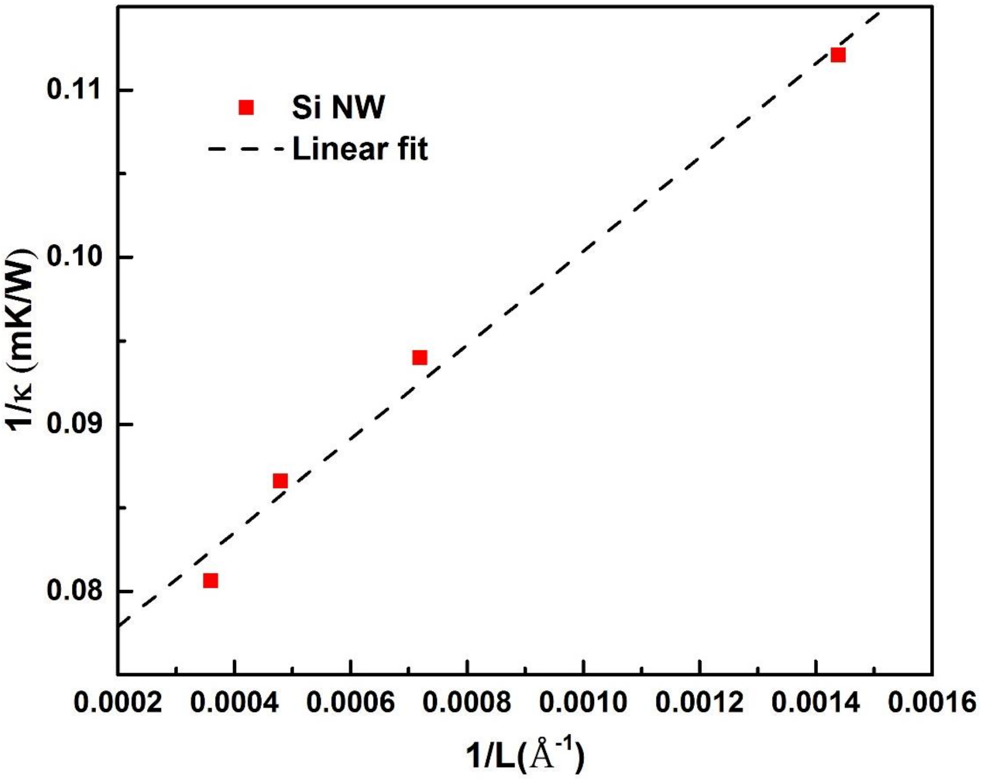

Figure S2. Thermal conductivity of regular Si/Ge SNW as a function of its period length (L_s). The black vertical dash line indicates the position of minimum thermal conductivity.

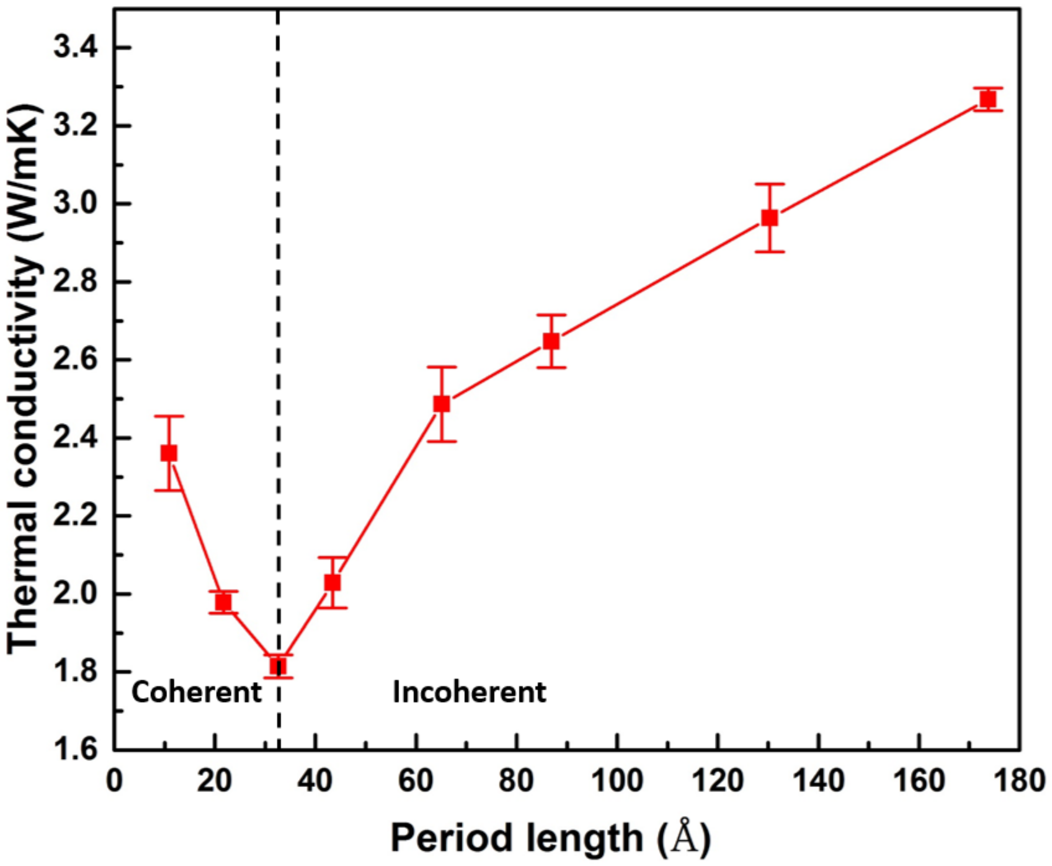

Figure S3. (a) Side view of the structure of regular Si/Ge SNW with period of "AB". (b) The frequency dependent CL of regular Si/Ge SNW with period of "AB". (c) The phonon DOS of regular Si/Ge SNW with period of "AB".

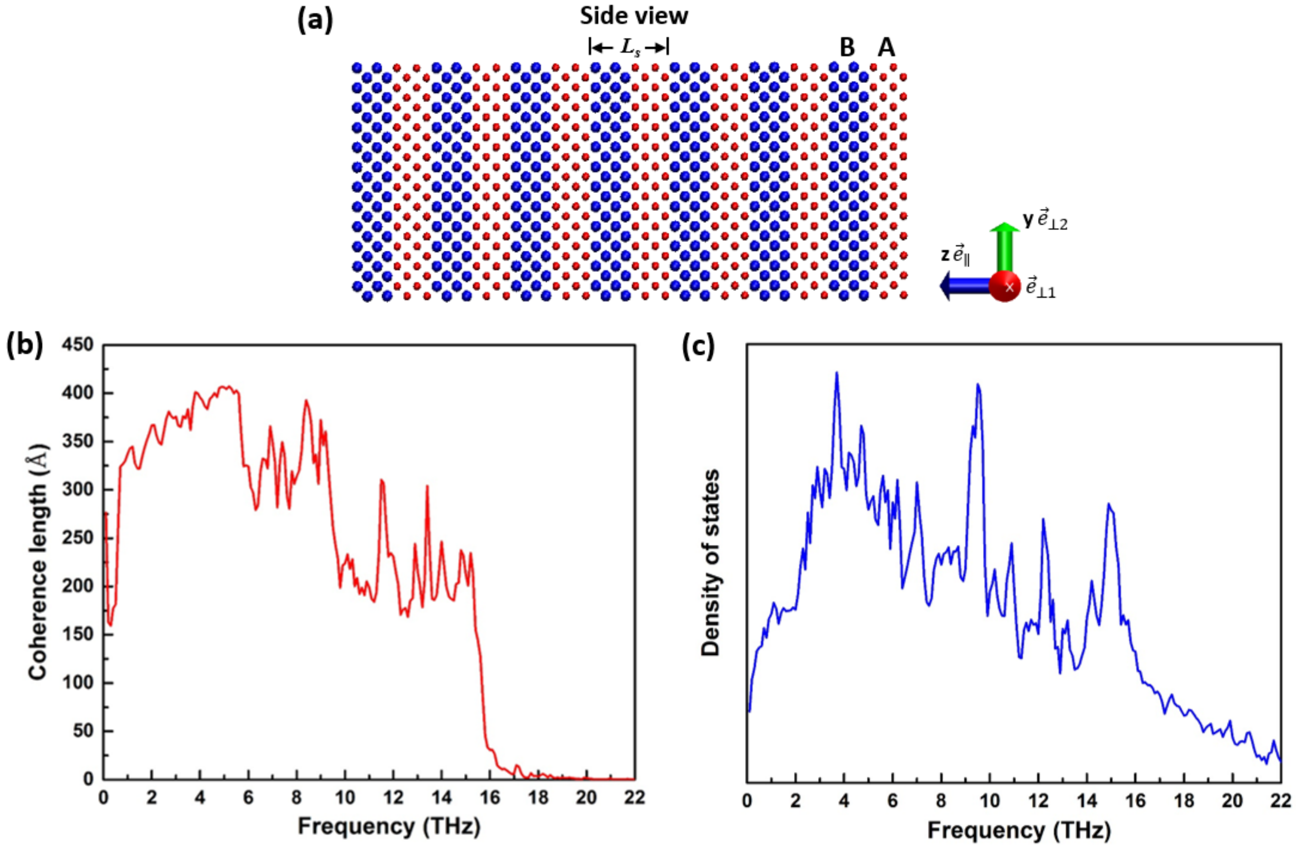

Supplement: Supporting Information [file srep16697-s1.pdf]
